# Asymptomatic bacteriospermia and infertility—what is the connection?

**DOI:** 10.1007/s15010-022-01828-5

**Published:** 2022-04-26

**Authors:** Yannic Volz, Benedikt Ebner, Paulo Pfitzinger, Elena Berg, Ekaterina Lellig, Julian Marcon, Matthias Trottmann, Armin Becker, Christian G. Stief, Giuseppe Magistro

**Affiliations:** 1 Department of Urology, Klinikum der Universität München, Munich, Germany; 2 Urologie und Andrologie am Promenadeplatz, Munich, Germany; 3 Department of Urology, Ludwig-Maximilians-University, Marchioninistrasse 15, 81377 Munich, Germany

**Keywords:** Urinary tract infection, Infertility, Bacteriospermia, Spermiogram

## Abstract

**Objective:**

To determine the impact of asymptomatic bacteriospermia on semen quality in subfertile men.

**Methods:**

We conducted a retrospective, single-centre cohort study in 1300 subfertile men. In those diagnosed with asymptomatic bacteriospermia we performed univariate and multivariate logistic regression models to evaluate the strain-specific association with semen parameters.

**Results:**

Asymptomatic bacteriospermia was diagnosed in 3.2% of patients. The microbiological semen analysis revealed a poly-microbial result in 60%. The most common bacterial species were *coagulase-negative Staphylococci species* (71.4%), *Streptococcus viridans* (50.0%) and *Enterococcus faecalis* (26.2%). Sexually transmitted pathogens were identified in 11.9% of semen samples. The detection of *Streptococcus viridians* or *Haemophilus parainfluenzae* correlated with impaired sperm morphology (*p* < 0.05). The presence of *coagulase-negative Staphylococci species* or *Enterococcus faecalis* was associated with pathological low counts of live spermatozoa (*p* < 0.05). In multivariate analysis only *Enterococcus faecalis* showed a significant impact on sperm concentration (OR 4.48; 95% CI 1.06–22.10; *p* = 0.041).

**Conclusions:**

Asymptomatic bacteriospermia has always been a subject of great controversy. There is still an ongoing debate whether to treat or not to treat. Here, we demonstrate that asymptomatic bacteriospermia is clearly associated with impaired semen quality. Our findings speak in favour of strain-specific interactions with semen parameters. Especially *Enterococcus faecalis* seriously affects sperm concentration.

## Introduction

Urogenital tract infections and inflammation have been shown to impact semen quality and are significant etiological factors for male infertility [1–3]. Around 6–44% of all male cases with infertility are reported to be caused by infection and/or inflammation [4, 5]. Acute and chronic infections and consequently ongoing inflammation may lead to a reduced sperm cell function and may even compromise the whole spermatogenic process [6, 7]. The most common underlying urinary tract infections include chronic urethritis, acute/chronic prostatitis, and scrotal infections, such as epididymitis or orchitis [8–10]. Even viral infections such as human immunodeficiency virus (HIV) have been associated with chronic inflammation and herewith caused infertility [11]. Although symptomatic infections with pathogens such as *Escherichia coli* (*E.coli*) or *Chlamydia trachomatis* have already been proven to be directly associated with male infertility [9, 10], it still remains unclear, whether bacterial detection in asymptomatic men is associated with male infertility. Data regarding its effect on sperm quality is highly heterogenic and contradictory [3]. Potential contamination of ejaculate samples with urethral commensals and the question on the most accurate diagnostic sample contribute to this unsolved issue [12]. Furthermore, it remains elusive what bacterial species could be relevant and whether antibiotic treatment is justified in asymptomatic men. Therefore, the main objective of this study is to determine the impact of asymptomatic bacteriospermia on semen quality in subfertile men.

## Materials and methods

### Study design

**Fig. 1:**
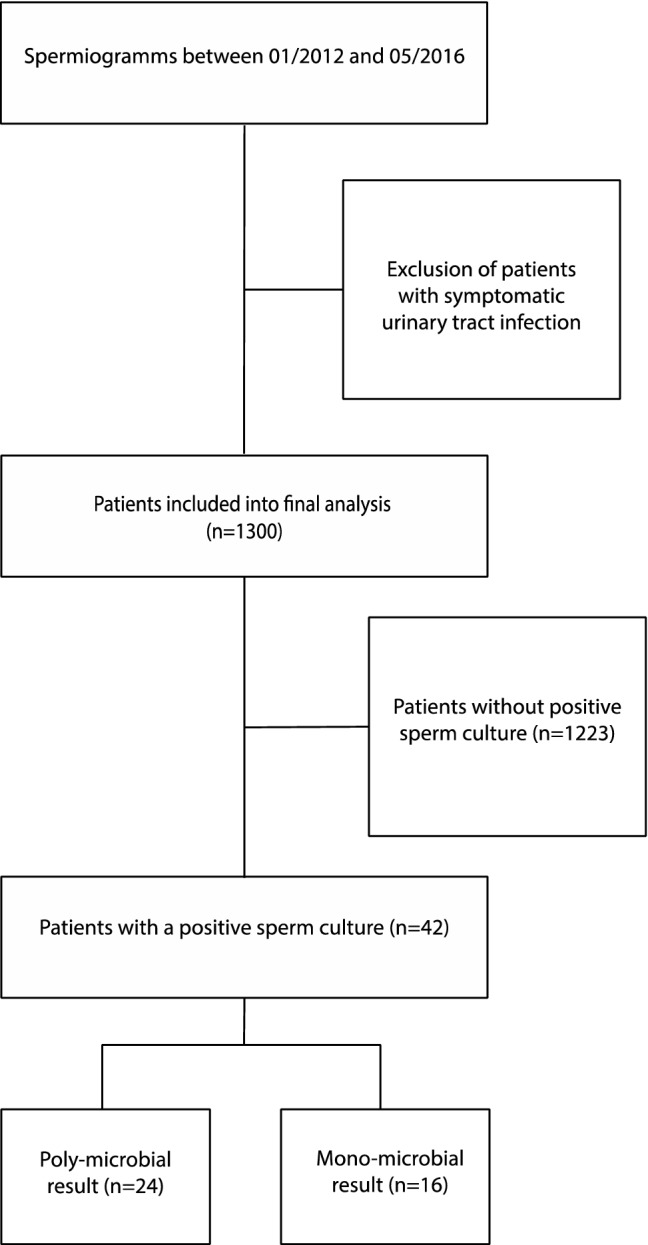
Patient selection

At our tertiary referral center we retrospectively evaluated semen analyses of men with suspected fertility disorder from January 2012 and May 2016. Patients with symptomatic urinary tract infection were excluded. We included 1300 spermiograms for the final analysis. Detailed information about the patient recruitment is displayed in Fig. 1.

### Semen collection and analysis

Semen samples were collected directly at the clinic into sterile containers after 3–5 days of sexual abstinence. To prevent contamination, the patients were asked to wash and disinfect hands, as well as the external genitalia. Semen analysis was performed within 1 h of collection according to current World Health Organisation (WHO) recommendations [13]. The following variables were obtained: Volume (in mL), concentration (10^6^ mL^−1^), total sperm number (10^6^/ejaculate), motility (%, A-rapid progressive motility, B-slow/sluggish progressive motility, C-no progressive motility, D-immotility), morphology (%), Vitality (%) and white blood cells (10^6^ mL^−1^). Variables were classified according to the 2010 version of the WHO laboratory manual for the examination and processing of human semen [13]. Of note, the new 2021 update is available, but it was not considered due to the earlier recruitment period of the current study.

### Microbiological diagnostics

We used ≥ 100 peroxidase-positive leucocytes per ml as the cutoff for subsequent microbiological testing based on standard culture techniques and strain-specific polymerase chain reactions (PCR) for sexually transmitted infections (STI). The STI–PCR addressed *Mycoplasme genitalium*, *Mycoplasma hominis*, *Ureaplasma urealyticum*, *Ureaplasma parvum, Chlamydia trachomatis* and *Neisseria gonorrhoea.* Bacteriospermia was defined as ≥ 10^3^ colony-forming units (CFU)/mL. Bacteriuria was excluded by standard urine culture.

### Statistical analysis

Data are expressed in mean and standard deviation in case of metric variables and number (%) in case of categorical variables. Statistical analysis was conducted to investigate any associations between a positive microbiological result and any pathological semen parameter according to WHO guidelines. Univariate analysis included Mann–Whitney *U* Test for metric variables and Chi^2^-Test for categorical variables. Multivariate analysis was performed using binary regression. A *p*-value < 0.05 was considered statistically significant and reported two-sided. All statistics were performed using SPSS Statistics v26 (SPSS, Chicago, IL, USA).

## Results

### Patient characteristics

Overall, 1300 consecutive semen analyses were included into the final evaluation. In 77 (5.9%) patients we identified at least 100 leucocytes/mL. However, among those only 42 (3.2%) showed a positive microbiological result. Previous urinalysis revealed no growth in all patients with bacteriospermia excluding a potential role of bacteriuria for microbiological findings in semen. Patient selection is displayed in Table 1. Mean overall patient age was 36.7 years (± 8.2). Patients diagnosed with asymptomatic bacteriospermia were significantly older than men with negative microbiological results (39.9 years vs. 37.0 years; *p* = 0.004).

**Table 1:** Overall patient and semen characteristics and microbiological results

	Total (*n* = 1300)	Culture negative (*n* = 35)	Culture positive (*n* = 42)	*p value*
Age [mean ± SD]	36.7 ± 8.2	37.0 ± 7.5	39.9 ± 10.4	**0.004**
*Spermiogram*				
Volume [mean ± SD]	3.3 ± 2.6	3.5 ± 1.9	2.9 ± 1.9	0.114
*WHO path. [n (%)]*				
Sperm/mL [mean ± SD]	52.1 ± 73.3	47.6 ± 49.1	41.5 ± 54.5	0.825
*WHO path. [n (%)]*				
Overall concentration (in mio.) [mean ± SD]	165.2 ± 273.7	140.8 ± 182.1	113.7 ± 153.3	0.925re
*WHO path. [n (%)]*				
Motility A % [mean ± SD]	9.6 ± 9.8	12.1 ± 9.6	10.0 ± 9.8	0.922
*WHO path. [n (%)]*				
Motility B % [mean ± SD]	24.4 ± 31.0	29.7 ± 11.0	27.7 ± 9.6	0.943
*WHO path. [n (%)]*				
Motility C % [mean ± SD]	14.8 ± 11.1	18.0 ± 6.1	17.6 ± 5.1	0.162
*WHO path. [n (%)]*				
Motility D % [mean ± SD]	30.3 ± 23.4	40.3 ± 21.1	44.7 ± 20.6	**0.001**
*WHO path. [n (%)]*				
Leucocytes (mio./mL)	0.1 ± 0.2	0.1 ± 0.2	0.3 ± 0.5	** < 0.001**
*WHO path. [n (%)]*				
Normal shape in % [mean ± SD]	5.7 ± 4.8	5.4 ± 2.5	4.5 ± 2.2	**0.018**
*WHO path. [n (%)]*				
Vitality in % [mean ± SD]	55.4 ± 14.0	53.9 ± 13.4	52.2 ± 14.4	0.053
*WHO path. [n (%)]*				
pH [mean ± SD]	8.1 ± 0.3	8.1 ± 0.3	8.2 ± 0.3	**0.019**
*WHO path. [n (%)]*				
Positive urine culture		0 (0)	0 (0)	
Sperm culture				
Poly-microbial [*n* (%)]		24 (60.0)		
Mono-microbial [*n* (%)]		16 (40.0)		
Microbial spectrum [*n* (%)]				
* Coagulase-negative Staphylococcus *spp.		30 (71.4)		
* Streptococcus viridans*		21 (50.0)		
* Enterococcus faecalis*		11 (26.2)		
* Hemophilus parainfluenzae*		6 (14.3)		
* Neisseria *spp.		5 (11.9)		
* Ureaplasma urealyticum*		4 (9.5)		
* Corynebacterium* spp.		3 (7.1)		
* Streptococcus agalactiae*		3 (7.1)		
* Chlamydia trachomatis*		1 (2.4)		
* Escherichia coli*		1 (2.4)		
* Non-hemolytic Streptococci*		1 (2.4)		
* Streptococcus anginosus*		1 (2.4)		
* Klebsiella pneumoniae*		1 (2.4)		

*p*-value < 0.05 was considered statistically significant (in bold)

### Semen analysis

Detailed information about the results of the semen analysis is depicted in Table 1. Men with asymptomatic bacteriospermia (*n* = 42) were compared to men with a negative microbiological result. Patients with asymptomatic bacteriospermia showed significantly more completely immotile (Motility D) spermatozoa (44.7% vs. 40.3%; *p* = 0.001), significantly more leucocytes (0.3 vs. 0.1; *p* < 0.001) and significantly less normal shaped spermatozoa (4.5 vs. 5.4; *p* = 0.0018). Samples with a positive bacterial result also demonstrated a slightly more basic pH-value, which was of statistical significance (8.2 vs. 8.1; *p* = 0.0019). Other variables such as volume, sperm/ml or overall concentration showed no significant differences.

### Microbial spectrum

**Fig. 2:**
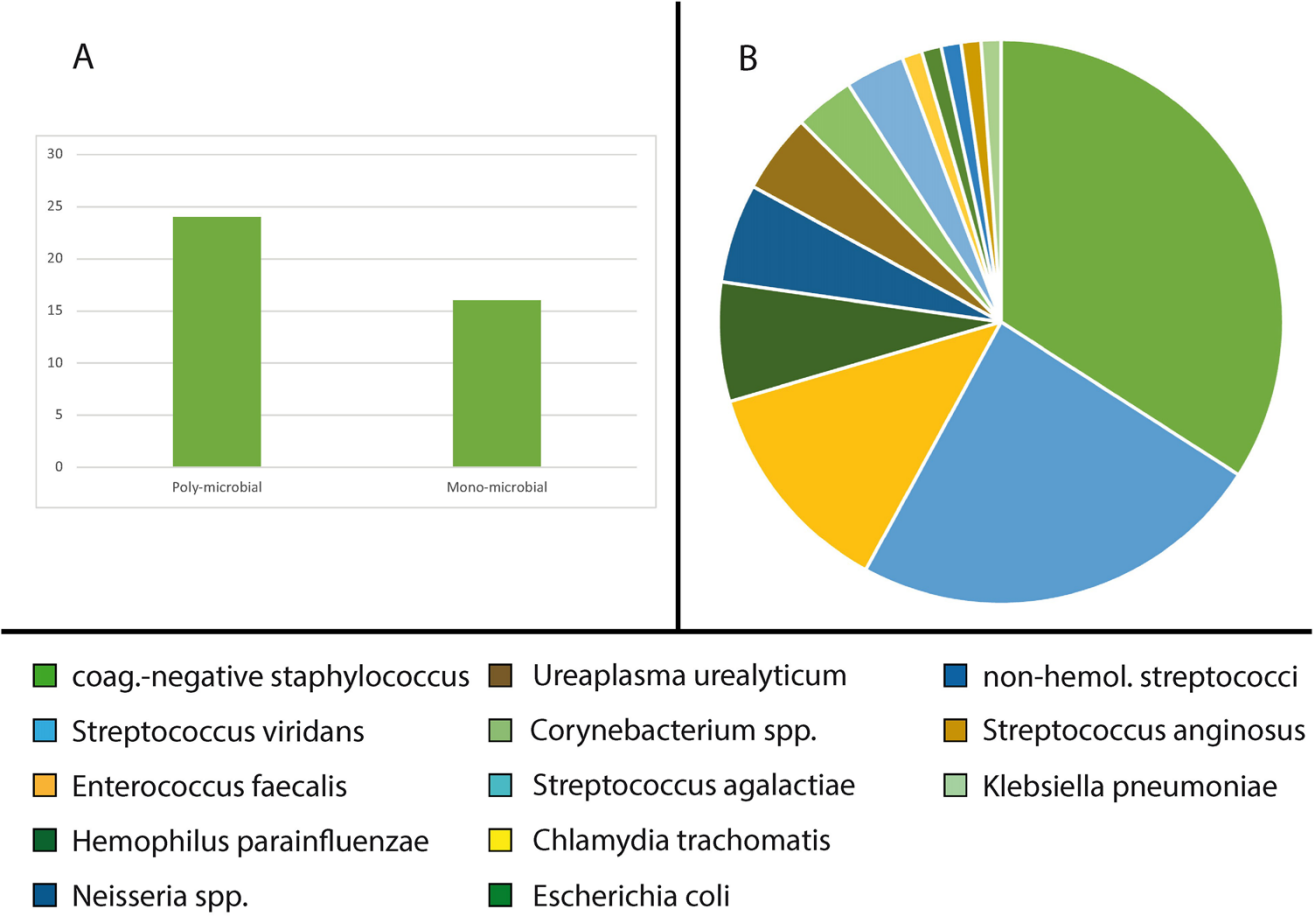
**A** Microbiological results (poly- vs. mono-microbial); **B** microbial spectrum

In 24 men with bacteriospermia (60.0%) we observed a poly-microbial result compared to 16 patients (40.0%) with a mono-microbial outcome (Table 1, Fig. 2A). The most frequent pathogens were *coagulase-negative Staphylococci species* (71.4%), *Streptococcus viridans* (50.0%) and *Enterococcus faecalis* (26.2%) (Table 1, Fig. 2B). Common sexually transmitted pathogens were found less frequently*. Ureaplasma urealyticum* was found in four patients (9.5%) and *Chlamydia trachomatis* in only one man (2.4%).

### Independent factors for pathologic spermiogram variables

**Fig. 3:**
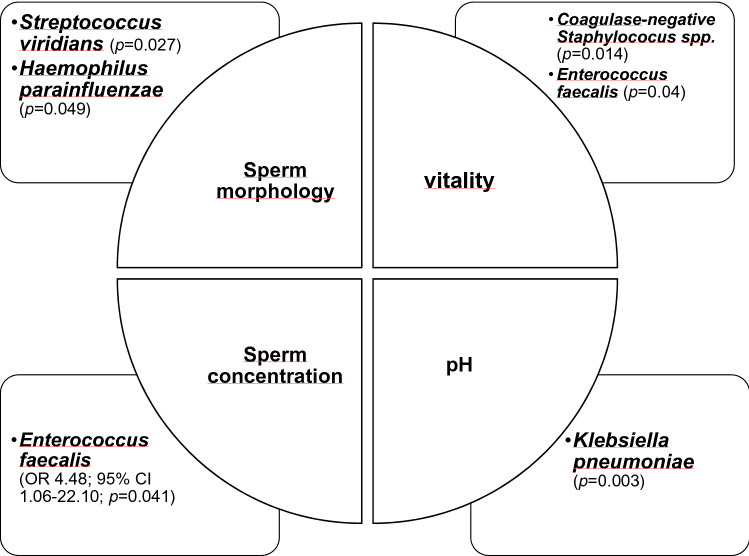
Strain-specific impact on semen parameters in uni- and multivariate logistic analysis

Finally, we performed an analysis to investigate whether asymptomatic bacteriospermia was associated with impaired semen parameters according to current WHO guidelines (Fig. 3). In univariate analysis *Enterococcus faecalis, Streptococcus viridans, Hemophilus parainfluenzae, Klebsiella pneumoniae* and *coagulase-negative Streptococcus* spp. showed a significant association with pathological changes. Interestingly, our results suggested a strain-specific impact. The detection of *Streptococcus viridians* or *Haemophilus parainfluenzae* correlated with impaired sperm morphology (*p* < 0.05). The presence of *coagulase-negative Staphylococci species* or *Enterococcus faecalis* was associated with pathological low counts of live spermatozoa (*p* < 0.05). However, after multivariate analysis only *Enterococcus faecalis* showed a significant impact on a reduced overall concentration of sperm (OR 4.48; 95% CI 1.06–22.10; *p* = 0.041).

## Discussion

Male accessory gland infection (MAGI) and inflammation have widely been discussed and proven to be a reason for reduced sperm quality and, therefore, infertility [1–3]. Infections and the herewith-caused inflammation can directly interefere with the spermatogenic process [6, 7]. So far, different types of urogenital tract infections have been linked with reduced sperm quality. Shang et al*.* published a meta-analysis of seven studies related to chronic bacterial prostatitis and semen quality and found that there was a significant negative effect on sperm vitality, sperm total motility and the number of progressively motile sperm [8]. Furthermore, uropathogenic *E. coli* (UPEC) have been shown by Lang et al*.* to reduce sperm count even after the successful treatment of UPEC-associated epididymitis [9]. Albeit, regarding asymptomatic infections and possibly contamination, the current literature is very contradictive. Mehta et al*.* reported the presence of aerobic cocci in around 50% of semen samples in infertile males [14]. These numbers are comparable to the current study, as most pathogens we found were also aerobic cocci. Mehta et al*.* isolated *Enterococcus faecalis* from around 50% of their samples. In the current study *Enterococcus faecalis* was found in around 26% of the samples.

Regarding the overall quality of the semen, the present study was able to reveal that there were significant differences between patients with a positive culture and patient with a negative culture despite elevated leucocyte count. These patients were shown to have significantly higher numbers of immotile sperm, significantly higher leucocyte numbers as well as significantly less normal shaped sperm. Similar results were demonstrated by Moretti et al. as they were able to show decreased motility in the group of patients with positive cultures, yet their study did not include all WHO variables used today [15]. The negative impact on motility in general is a well-documented fact in recent literature [16]. It is notable, that patients with positive cultures also had higher values of leucocytes within their sperm. This could act as a confounder, as there seems to be a negative correlation between the leucocyte levels and sperm concentration and motility as well as sperm morphology [17]. Therefore, it could be argued that not the presence of the bacteria alone might impact sperm quality. Still it remains controversial whether there is a clinical significance of increased leucocytes in the ejaculate as leucocytospermia is not necessarily associated with bacteria-induced inflammation [11].

Furthermore, the present study suggested that *Enterococcus faecalis* may be considered as an independent predictor for a reduced overall sperm concentration according to current WHO cut-off values [13]. Interestingly, Moretti et al. were able to show that there was an increased number of infertile patients that also were positive for *Enterococcus faecalis*. Correspondingly, Villegas et al*.* were able to demonstrate in an in vitro study that *Enterococcus faecalis* induced apoptosis in human sperm possibly either by direct cytotoxic activity and/or the contact with flagella and pili [18]. This could be one way this pathogen could lead to the decreased overall sperm concentration. Yet, a lot of different pathogenic mechanisms have been proposed by other groups on how bacteria could possibly damage spermatozoa. The presence of *Ureaplasma urealyticum* for example leads to decreased microelements, such as zinc and selenium, which consequently reduces the capability to withstand oxidative stress [16]. In addition, cross-reaction between antigens on species such as *Enterococcus faecalis* or *E. coli* have been discussed as possible pathomechanism, as they could lead to antibody production that damage the flagella of the spermatozoa [15].

The current study demonstrated that an asymptomatic infection with *Enterococcus faecalis* may lead to a higher risk of reduced overall sperm concentration. Another question that arises concerns the benefit of antimicrobial treatment. In case of sexually transmitted diseases antimicrobial therapy is always necessary and should be performed according to current guidelines [19, 20]. Yet, antimicrobial treatment does not necessarily lead to increased fertility as it has been shown In case of epididymitis or orchitis [11, 21]. On the other hand inflammation itself plays an important role and has, therefore, been discussed as a possible treatment approach [22].

Overall, our results suggest that asymptomatic bacterial infection in infertile patients can lead to decreased motility, a reduced number of normal shaped spermatozoa as well as increased leucocytes. Furthermore, we were able to show that the presence of *Enterococcus faecalis* can negatively impact the overall sperm concentration.

However, this study is not devoid of limitations. First, it is a mono-centre, retrospective study with the inherent shortcomings. Second, we did not know whether the infertile men had a previous history of urogenital tract infections, as we only excluded patients that currently reported symptoms associated with urinary tract infection or MAGI. Finally, we proved that there is a strong strain-specific connection between asymptomatic bacteriospermia and impaired semen quality. However, the decisive question is whether targeted antimicrobial treatment might improve those affected parameters and consequently might result in successful conception. This issue of clinical importance warrants further investigation in prospective randomized trials.

## Conclusions

In the current study, we provided evidence that asymptomatic bacteriospermia in subfertile men lead to significantly reduced spermatozoa motility and decreased the number of normal shaped spermatozoa. In addition, to our knowledge, we are the first to show that asymptomatic *Enterococcus faecalis* infection may significantly impact overall sperm concentration and can cause a decrease in overall sperm concentration. We need additional clinical trials to evaluate the clinical benefit of antimicrobial treatment for this scenario.

## References

[1] Fijak M, Pilatz A, Hedger MP, Nicolas N, Bhushan S, Michel V (2018). Infectious, inflammatory and ‘autoimmune’male factor infertility: how do rodent models inform clinical practice?. Hum Reprod Update.

[2] Weng S-L, Chiu C-M, Lin F-M, Huang W-C, Liang C, Yang T (2014). Bacterial communities in semen from men of infertile couples: metagenomic sequencing reveals relationships of seminal microbiota to semen quality. PloS one..

[3] Gimenes F, Souza RP, Bento JC, Teixeira JJ, Maria-Engler SS, Bonini MG (2014). Male infertility: a public health issue caused by sexually transmitted pathogens. Nat Rev Urol.

[4] Comhaire F (1987). Towards more objectivity in the management of male infertility. The need for a standardized approach. Int J Androl.

[5] Han H, Liu S, Zhou XG, Tian L, Zhang XD (2016). Aetiology of obstructive azoospermia in Chinese infertility patients. Andrologia.

[6] Henkel R, Schill WB (1998). Sperm separation in patients with urogenital infections. Andrologia.

[7] Urata K, Narahara H, Tanaka Y, Egashira T, Takayama F, Miyakawa I (2001). Effect of endotoxin-induced reactive oxygen species on sperm motility. Fertil Steril.

[8] Shang Y, Liu C, Cui D, Han G, Yi S (2014). The effect of chronic bacterial prostatitis on semen quality in adult men: a meta-analysis of case-control studies. Sci Rep.

[9] Lang T, Dechant M, Sanchez V, Wistuba J, Boiani M, Pilatz A (2013). Structural and functional integrity of spermatozoa is compromised as a consequence of acute uropathogenic *E. coli*-associated epididymitis. Biol Reprod..

[10] Schuppe HC, Pilatz A, Hossain H, Diemer T, Wagenlehner F, Weidner W (2017). Urogenital infection as a risk factor for male infertility. Dtsch Arztebl Int.

[11] Rusz A, Pilatz A, Wagenlehner F, Linn T, Diemer T, Schuppe HC (2012). Influence of urogenital infections and inflammation on semen quality and male fertility. World J Urol.

[12] Cottell E, Harrison RF, McCaffrey M, Walsh T, Mallon E, Barry-Kinsella C (2000). Are seminal fluid microorganisms of significance or merely contaminants?. Fertil Steril.

[13] WHO laboratory manual for the examination and processing of human semen (2010) 21243747

[14] Mehta RH, Sridhar H, Vijay Kumar BR, Anand Kumar TC (2002). High incidence of oligozoospermia and teratozoospermia in human semen infected with the aerobic bacterium *Streptococcus faecalis*. Reprod Biomed Online.

[15] Moretti E, Capitani S, Figura N, Pammolli A, Federico MG, Giannerini V (2009). The presence of bacteria species in semen and sperm quality. J Assist Reprod Genet.

[16] Fraczek M, Szumala-Kakol A, Jedrzejczak P, Kamieniczna M, Kurpisz M (2007). Bacteria trigger oxygen radical release and sperm lipid peroxidation in in vitro model of semen inflammation. Fertil Steril.

[17] Henkel R, Maass G, Hajimohammad M, Menkveld R, Stalf T, Villegas J (2003). Urogenital inflammation: changes of leucocytes and ROS. Andrologia.

[18] Villegas J, Schulz M, Soto L, Sanchez R (2005). Bacteria induce expression of apoptosis in human spermatozoa. Apoptosis.

[19] Workowski KA, Bolan GA (2015). Sexually transmitted diseases treatment guidelines, 2015. MMWR Recomm Rep..

[20] Wagenlehner FM, Brockmeyer NH, Discher T, Friese K, Wichelhaus TA (2016). The presentation, diagnosis, and treatment of sexually transmitted infections. Dtsch Arztebl Int.

[21] Pilatz A, Wagenlehner F, Bschleipfer T, Schuppe H-C, Diemer T, Linn T (2013). Acute epididymitis in ultrasound: results of a prospective study with baseline and follow-up investigations in 134 patients. Eur J Radiol.

[22] Lackner JE, Herwig R, Schmidbauer J, Schatzl G, Kratzik C, Marberger M (2006). Correlation of leukocytospermia with clinical infection and the positive effect of antiinflammatory treatment on semen quality. Fertil Steril.

